# The effects of heating on the physicochemical properties of tricalcium silicate root canal sealers

**DOI:** 10.1590/0103-6440202305237

**Published:** 2023-10-27

**Authors:** Lincon Hideo Nomura, Eduardo Antunes Bortoluzzi, Franklin R. Tay, Lucas da Fonseca Roberti Garcia, Cleonice da Silveira Teixeira

**Affiliations:** 1 Department of Dentistry, Federal University of Santa Catarina (UFSC), Florianópolis, SC, Brazil; 2 Department of Diagnosis & Oral Health, Division of Endodontics, University of Louisville, Louisville, KY, USA.; 3 Department of Endodontics, The Dental College of Georgia, Augusta University, GA, USA.

**Keywords:** tricalcium silicate, dental sealers, epoxy resin, physicochemical properties, root canal sealer

## Abstract

This study evaluated the effect of heating on the physicochemical properties and surface changes of tricalcium silicate sealers. Three tricalcium silicate root canal sealers (Bio-C Sealer, BioRoot-RCS, EndoSequence BC Sealer), and one epoxy resin-based sealer (AH Plus; control) were tested. The effect of heating on setting time (ST) and flowability were assessed according to ANSI/ADA 57 and ISO 6876 standards. Solubility and dimensional change (DC) of the set sealers were evaluated at 24 hours and after 30 days; the pH of the water used in the DC testing was also measured. Tests were repeated with heated sealers in an oven at 100 °C for 1 min. SEM and EDS analysis were performed. Data were analyzed using One-Way ANOVA and Tukey post-hoc tests (α=5%). Heating decreased the ST for AH Plus and EndoSequence (p<0.05). Heating reduced flowability (p<0.05) and increased pH for AH Plus (p<0.05). The solubility of Bio-C (dried specimens) was not in accordance with the ANSI/ADA standard. The solubility of EndoSequence was significantly higher (p<0.05) when it was heated and dried after 30 days. DC of Bio-C (24 h and 30 days), BioRoot-RCS (30 days) and AH Plus (24 h and 30 days) were not in accordance with the standards. SEM and EDS analysis showed significant changes in sealer microstructure after heating. In conclusion, heating decreased the ST and increased the solubility of EndoSequence BC sealer. No significant changes in flowability, DC, and pH were identified for all three tricalcium silicate sealers after heat application. However, all sealers had significant surface changes.

## Resumo

Este estudo avaliou o efeito do aquecimento nas propriedades físico-químicas e nas alterações superficiais de cimentos de silicato tricálcico. Três cimentos endodônticos de silicato tricálcico (Bio-C Sealer, BioRoot-RCS, EndoSequence BC Sealer) e um cimento à base de resina epóxi (AH Plus) foram testados para avaliar o efeito do aquecimento no tempo de presa (TP) e escoamento de acordo com as normas ANSI/ADA 57 e ISO 6876. A solubilidade e alteração dimensional (AD) dos cimentos foram avaliadas após 24 horas e 30 dias. O pH da água usada no teste de AD também foi medido. Os testes foram repetidos com cimentos aquecidos em um forno a 100 °C por 1 min. Análises em MEV e EDS foram realizadas. Os dados foram analisados pelos testes de ANOVA-1 via e *post hoc* de Tukey (α=5%). O aquecimento diminuiu o TP para AH Plus e EndoSequence (p<0,05). O aquecimento reduziu o escoamento (p<0,05) e aumentou o pH do AH Plus (p<0,05). A solubilidade do Bio-C (amostras desidratadas) não estava de acordo com o padrão ANSI/ADA. A solubilidade da EndoSequence foi significativamente maior (p<0,05) quando aquecida e desidratada após 30 dias. As AD de Bio-C (24 h e 30 dias), BioRoot-RCS (30 dias) e AH Plus (24 h e 30 dias) não estavam de acordo com as normas ADA e ISO. A análise em MEV e EDS mostrou alterações significativas na estrutura dos cimentos após aquecimento. Em conclusão, o aquecimento diminuiu o TP e aumentou a solubilidade do cimento EndoSequence BC. Não foram identificadas mudanças significativas no escoamento, AD e pH para os cimentos de silicato tricálcico após aquecimento. Porém, todos os cimentos tiveram alterações significativas na superfície.

## Introduction

Warm vertical compaction of thermoplastic materials has been advocated for improving the adaptation of root fillings in canal spaces with complex canal anatomy ^1^ ^,^ ^2^. During the warm vertical compaction technique, heat is applied in continuous waves, and the full procedure usually lasts a minute ^3^. The heat transmitted by a metal carrier to the gutta-percha also warms up the root canal sealer ^4^.

It is known that heating may alter the physical and chemical properties of sealers ^3^ ^,^ ^4^ ^,^ ^5^ ^,^ ^6^. The heating of an epoxy resin-based sealer to a temperature higher than the recommended temperature range, or for a duration longer than 30 s, resulted in degradation of the epoxy resin chemical structure ^5^. The same experiment also evaluated the effect of heat application on a tricalcium silicate sealer (BioRoot-RCS, Septodont, Saint-Maur-des-Fossés, France) and found no chemical alteration associated with heating apart from the loss of water through evaporation ^5^. The effect of heating on other tricalcium silicate-based sealers has been reported ^4^ ^,^ ^6^ ^,^ ^7^. For instance, the setting time of iRoot sealer (Innovative BioCeramix Inc., Burnaby, British Columbia, Canada) was significantly shortened and its flow was reduced when heated to 140 ^o^C ^7^. The iRoot sealer is marketed under other tradenames: Endosequence BC sealer, (Brasseler USA, Savannah, GA, USA), Edge-Endo Bioceramic Sealer (EdgeEndo, Albuquerque, NM, USA), and TotalFill BC Sealer (FKG Dentaire, La Chaux-de-Fonds, Switzerland). Another tricalcium silicate sealer, Bio-C Sealer (Angelus, Londrina, Brazil) is an injectable and ready-to-use sealer, which contains tricalcium silicate, tricalcium aluminate, calcium oxide, zirconium oxide, iron oxide, silicon dioxide and a dispersing agent ^8^. When compared with an epoxy resin-based (AH Plus; Dentsply Sirona, York, PA), Bio-C sealer had a shorter setting time, an alkaline pH, adequate flow, optimal radiopacity, and lower volumetric shrinkage ^8^. However, when heated to 100^o^C, Bio-C Sealer had its flowability reduced and its setting time significantly prolonged ^6^.

When choosing an endodontic sealer, proper physical, chemical, and biological properties are crucial to achieve a successful root canal treatment ^8^. Among these properties, the setting time allows adequate working time, leading to favorable handling throughout the root canal system obturation ^9^. Proper flowability allows the sealer to fill hard-to-reach areas, such as isthmuses and accessory canals ^10^. Conversely, the solubility of an endodontic sealer is undesirable, since its dissolution over time allows bacterial leakage and compromises root canal sealing ^11^. Similarly, an inappropriate dimensional change may favor the appearance of voids in the sealer/dentin and sealer/gutta-percha interfaces ^12^. Furthermore, an alkaline pH potentiates the antimicrobial effect of the endodontic sealer, and directly enhances its tissue repair capacity ^11^ ^,^ ^13^.

It has been demonstrated that heating of tricalcium silicate based-sealers leads to changes in their physicochemical properties, especially when submitted to the heat as occurs during thermoplastic obturation techniques ^3^ ^,^ ^4^ ^,^ ^5^ ^,^ ^7^. However, a deeper investigation into the effects of heating on this type of endodontic sealer is still necessary to consolidate previous findings ^7^.

Therefore, the purpose of this study was to evaluate the effect of heating on the physicochemical properties and surface changes of tricalcium silicate sealers. The null hypothesis tested was that heating would not affect the properties assessed, regardless of the sealer tested.

## Materials and methods

This is a material testing laboratory study, that verified the effect of heating on the physical and chemical properties of three tricalcium silicate sealers (Bio-C Sealer, BioRoot-RCS, and EndoSequence BC Sealer), using an epoxy resin-based sealer (AH Plus) as control. In addition, the surface characteristics of the sealers before and after heating were assessed under Scanning Electron Microscopy (SEM) and Energy-Dispersive X-ray Spectroscopy (EDS). The local Research Ethics Committee has confirmed that no ethical approval is required.

The composition of the four sealers is summarized in Box 1. Setting time and flow were evaluated according to the ADA standard 57 (2000) ^14^ and ISO 6876 (2012) ^15^ recommendations. The method proposed by Carvalho-Júnior et al*.* (2007) ^12^ was used for the evaluation of sealer solubility and dimensional change.

**Box 1:**
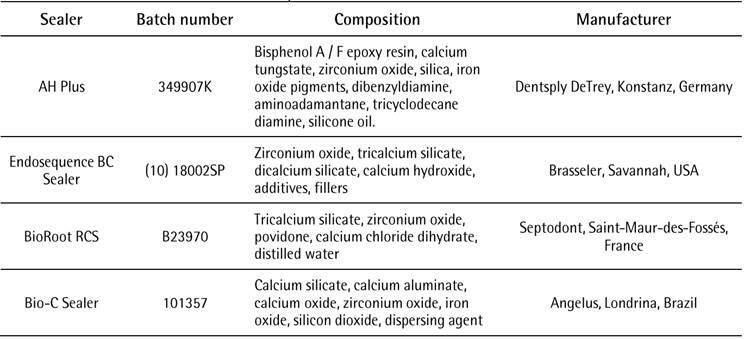
Endodontic sealers, lot number, composition and manufacturers.

### Physicochemical properties evaluation

For all tests, each endodontic sealer was mixed according to the manufacturer's instructions. The mixed sealers were distributed into two subgroups. In subgroup 1 (unheated sealers), they were stored in an incubator at 37 °C and 95% relative humidity. In subgroup 2 (heated sealers), they were placed in an oven (ECB2, Odontobrás, Ribeirão Preto, SP, Brazil) at 100 °C for 1 min. The temperature was constantly checked with a thermometer (ACC001CFC, Thermco Products Inc., Lafayette, NJ, USA). The thermometer bulb was coupled to an orifice at the top of the oven, with a display visible outside of it, which allowed us to verify and control the temperature throughout the experiment.

### Setting time

After mixing, the sealers were placed inside disk-shaped cavities (10 mm internal diameter, 1 mm high) created in plaster molds (Type II, K Dent, Quimidrol, Joinville, Brazil) that were previously immersed in water for 24 h at 37^o^C. The mixed sealers were distributed into two subgroups (n=3) and the heating process was performed as previously described. A 100 g Gilmore needle with a 2 mm diameter cylindrical tip was placed vertically on the surface of the sealers. Setting time was taken to be the time from the commencement of mixing (for AH Plus and BioRoot-RCS) or immediately after dispensing from the syringe (for premixed Bio-C and EndoSequence BC Sealer), to the time when needle indentation was no longer observed. ANSI/ADA 57 ^14^ states a maximum of 10% variation on the setting time of a sealer to what was established by the manufacturer.

### Flowability

After mixing for 180 s (AH Plus and BioRoot-RCS) or immediately after dispensing them (premixed sealers), 0.05 mL of each sealer was placed on a glass plate (40 x 40 x 5 mm). A 20 g glass plate and a load of 100 g were carefully applied on the top of each sealer. The samples were distributed into two subgroups (n=3) and the heating process was performed as previously described. The load was removed after 10 min and the major and minor diameters of each compressed sealer were measured using a pair of digital calipers. ISO 6876 ^15^ recommends a minimum diameter of 17 mm.

### Solubility

Teflon rings (7.75 mm internal diameter, 1.5 mm thick) were used to fabricate the sealers’ samples ^12^. The samples were distributed into two subgroups (n=6) and the heating process was performed as previously described. Heated and unheated samples were stored at 37 °C and 95% relative humidity for three-fold the setting time of each material (previously determined). The weight of each sample was first recorded and reweighed after 24 h of storage in a silica-containing desiccator. Each sample was individually placed inside a plastic container containing 7.5 mL of distilled water and returned to the oven at 37 °C. After 30 days, all samples were removed from their respective containers and reweighed after removal of excess water. Each sample was returned to the desiccator for 24 h and reweighed. The solubility of each sealer (hydrated and dehydrated samples), corresponding to the weight loss, was expressed as the percentage of weight lost over the original weight. Specification N^o^ 57 of ANSI/ADA ^14^ states that the maximum sealer solubility should be less than 3%.

### Dimensional Change

A Teflon mold containing drilled holes (3.58 mm high, 3.0 mm diameter) was used to fabricate cylindrical samples of each sealer (heated and unheated) for the 24-hour and 30-day tests. The samples (mixed sealer inside the Teflon mold) were distributed into two subgroups (n=6) for each test period, and the heating process was performed as previously described. Then, the samples were stored at 37 °C and 95% relative humidity for 24 h. Set samples were polished wet with 600-grit silicon carbide papers. After measuring the initial sample length on the longest axis of the cylinder (C), each sample was placed in a container with 2.24 mL of distilled water and stored at 37 ºC for 24 hours or 30 days. New measurements were performed after the removal of excess water from the retrieved aged samples. The dimensional change was calculated using the formula: [(C_1_ - C)/C] x 100, where C_1_ = sample length on the longest axis of the cylinder after 24 h or 30 days. Specification N^o^ 57 of ANSI/ADA ^14^ stablishes 0.1% maximum values for expansion and 1.0% for contraction.

### pH value

The pH of water from containers used in the dimensional change test was measured (24 h and 30 days) at room temperature using a pHmeter calibrated with buffer solution (pH 7).

### SEM and EDS Analysis

To evaluate the surface changes promoted by heating, SEM and EDS analysis were performed. One sample from each sealer (heated and unheated) was prepared. Metal rings (10-mm internal diameter x 1-mm height) were used to fabricate the samples. The sealers were mixed (AH Plus and BioRoot-RCS) according to their manufacturer’s instructions and placed inside the metal rings positioned on a glass plate. The premixed sealers (Bio-C and EndoSequence BC Sealer) were placed directly. The samples (metal rings filled with the respective sealers) were distributed into two subgroups and the heating process was performed as previously described. Heated and unheated specimens were stored in an oven at 37 °C and 95% relative humidity for three times the setting time of each sealer. Then, the specimens were fixed on metal cylindrical bases (stubs). Before the scanning electron microscope analysis, the specimens were dried, placed in a vacuum chamber, and sputter coated with a gold/palladium layer of approximately 300 A° (Bal-Tec SCD 005, Bal-Tec Co., USA). The analysis was performed under a scanning electron microscope (JEOL JSM 6390 LV, Akishima, Japan) operating at 10.0-20.0 kV (SCD 005, Bal-Tec Co., USA), at ×500, ×1,000, and ×2,000 magnifications. The geometric center of the surface of the sealer was standardized as the viewing area. The alterations on the sealers’ surface were qualitatively assessed by a blinded and previously calibrated examiner. Additional analysis was performed with Energy-Dispersive X-ray Spectroscopy (EDS).

### Statistical analysis

Each data set was tested for its normality (Shapiro-Wilk test) and homoscedasticity (modified Levene test) assumptions before the use of parametric statistical methods. One factor analysis of variance was used to set the difference among groups. The Tukey test was used for post-hoc pairwise comparisons. Statistical significance was set at α = 0.05. All data were tabulated and analyzed using the IBM Statistical Package for Social Sciences statistical program (IBM SPSS Statistics for Windows, Version 21.0, IBM Corp., Armonk, NY, USA).

## Results

**Figure 1:**
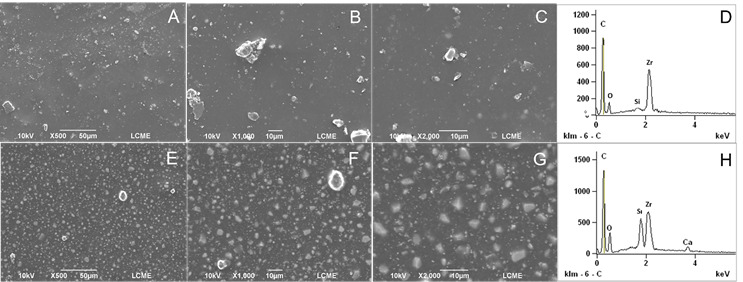
SEM and EDS analysis of AH Plus. A-C: Unheated AH Plus. Representative micrographs (×500 to ×2000 magnifications) under SEM analysis. D: Unheated AH Plus under EDS analysis. E-G: AH Plus heated to 100ºC for 1 minute (×500 to ×2000 magnifications under SEM analysis). It was possible to observe greater surface changes in the heated samples, with crystals deposition. H: AH Plus heated to 100ºC for 1 minute under EDS analysis. Note the higher peaks of silicon and calcium in the heated samples.

**Figure 2:**
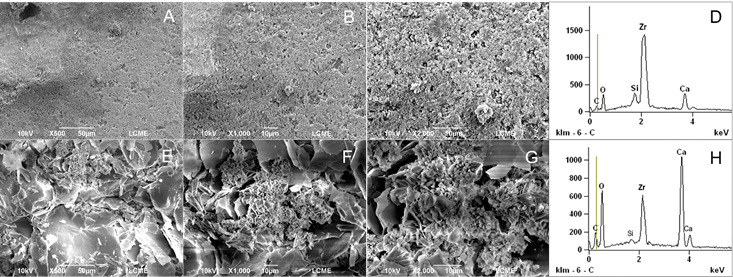
SEM and EDS analysis of EndoSequence. A-C: Unheated EndoSequence. Representative micrographs (×500 to ×2000 magnifications) under SEM analysis. D: Unheated EndoSequence under EDS analysis. E-G: EndoSequence heated to 100ºC for 1 minute (×500 to ×2000 magnifications under SEM analysis). Observe the formation of calcium hydroxide crystals similar to flower petals on a rough surface. H: EndoSequence heated to 100ºC for 1 minute under EDS analysis. Note the increase in calcium peaks and the reduction in zirconia and silicon peaks after heating.

**Figure 3:**
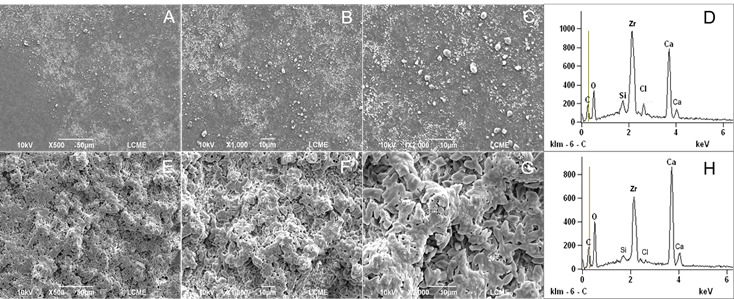
SEM and EDS analysis of BioRoot-RCS. A-C: Unheated BioRoot-RCS. Representative micrographs (×500 to ×2000 magnifications) under SEM analysis. D: Unheated BioRoot-RCS under EDS analysis. E-G: BioRoot-RCS heated to 100ºC for 1 minute (×500 to ×2000 magnifications under SEM analysis). The formation of a surface covered by glomeruli-like structures was more evident after the sealer is heating. h: BioRoot-RCS heated to 100ºC for 1 minute under EDS analysis. Moderate increase in the calcium peaks, and a considerable reduction in the zirconia and silica peaks.

**Figure 4:**
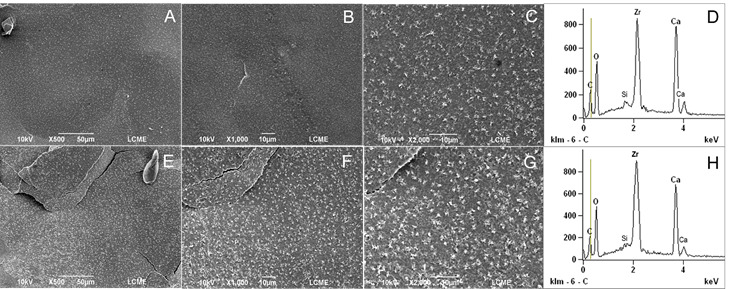
SEM and EDS analysis of Bio-C. A-C: Unheated Bio-C. Representative micrographs (×500 to ×2000 magnifications) under SEM analysis. D: Unheated Bio-C under EDS analysis. E-G: Bio-C heated to 100ºC for 1 minute (×500 to ×2000 magnifications under SEM analysis). Note the changes on the surface of heated samples, with greater deposition of sealer particles. H: Bio-C heated to 100ºC for 1 minute under EDS analysis, with similar results to the unheated Bio-C sealer.

Table 1 (setting time, flowability, and solubility tests), Table 2 (dimensional change and pH tests), and Figures 1,2,3,4(SEM and EDS analysis) demonstrate the results of the present study, which are detailed below.

**Table 1:** Mean values and standard deviations (SD) of the results obtained in the setting time (in minutes), flow (in millimeters), and solubility tests (%) of the different sealers, with and without heating.

Sealer	Setting Time (min)		Flow (mm)		Solubility (hydrated) (30 d)		Solubility (non-hydrated) (30 d)	
	Unheated	Heated	Unheated	Heated	Unheated	Heated	Unheated	Heated
	Mean ± SD	Mean ± SD	Mean ± SD	Mean ± SD	% ± SD	% ± SD	% ± SD	% ± SD
AH Plus	1127 ± 38^aA^	910 ± 16^aB^	24.1 ± 0.6^aA^	21.0 ± 1.8^aB^	0.070 ± 0.083^aA^	0.070 ± 0.060^aA^	0.071 ± 0.064^aA^	0.070 ± 0.034^aA^
EndoSequence	604 ± 48^bA^	489 ± 49^bB^	19.4 ± 0.3^bA^	19.9 ± 1.2^aA^	-0.529 ± 0.329^bA^	-0.533 ± 0.180^bA^	-1.101 ± 1.274^bA^	-2.083 ± 0.241^bB^
BioRoot-RCS	85 ± 5^cA^	67 ± 4^cA^	16.5 ± 0.5^cA^	16.3 ± 0.6^bA^	-1.269 ± 0.164^cA^	-0.980 ± 0.130^cA^	-2.528 ± 0.239^cA^	-2.289 ± 0.153^bA^
Bio-C	140 ± 23^cA^	124 ± 14^cA^	19.4 ± 0.2^bA^	18.5 ± 1.0^bA^	-1.834 ± 0.575^dB^	-1.091 ± 0.353^cA^	-5.524 ± 0.216^dA^	-5.215 ± 0.231^cA^
ISO6876/ANSI57	/	/	> 17 mm	> 17 mm	Less than -3%	Less than -3%	Less than -3%	Less than -3%

* Different upper-case letters between groups in the row and lower-case letters in the column represent a statistically significant difference (ANOVA and Tukey's test, α = 0.05).

** Negative values indicate a mass loss.

**Table 2:** Mean values and standard deviations (SD) of the Dimensional Change (%) and pH values, after 24 h and 30 days, of the different sealers, with and without heating.

Sealer	Dimensional Change (24 h)		Dimensional Change (30 d)		pH (24 h)		pH (30 d)	
	Unheated	Heated	Unheated	Heated	Unheated	Heated	Unheated	Heated
	Mean ± SD	Mean ± SD	Mean ± SD	Mean ± SD	% ± SD	% ± SD	% ± SD	% ± SD
AH Plus	0.19 ± 0.15^cA^	0.32 ± 0.20^aA^	0.43 ± 0.40^aA^	0.55 ± 0.24^aA^	6.85 ± 0.27^bB^	7.26 ± 0.09^bA^	7.00 ± 0.22^cA^	7.06 ± 0.11^bA^
EndoSequence	-0.25 ± 0.45^bA^	-0.24 ± 0.21^bA^	-0.20 ± 0.50^bA^	-0.14 ± 0.16^bA^	12.19 ± 0.06^aA^	12.21 ± 0.16^aA^	12.58 ± 0.03^aA^	12.32 ± 0.61^aA^
BioRoot-RCS	-0.10 ± 0.15^cA^	-0.10 ± 0.15^cA^	0.10 ± 0.15^cA^	0.15 ± 0.25^cA^	12.16 ± 0.02^aA^	12.17 ± 0.02^aA^	12.01 ± 0.14^bA^	12.19 ± 0.09^aA^
Bio-C	0.34 ± 0.12^aA^	0.36 ± 0.70^aA^	0.29 ± 0.19^cA^	0.31 ± 0.71^cA^	12.20 ± 0.05^aA^	12.27 ± 0.08^aA^	12.51 ± 0.05^aA^	12.39 ± 0.16^aA^
ANSI/ADA 57	< +0.1/-1.0	< +0.1/-1.0	< +0.1/-1.0	< +0.1/-1.0	/	/	/	/

* Different upper-case letters between groups in the row and lower-case letters in the column represent a statistically significant difference (ANOVA and Tukey's test, α = 0.05).

** Negative values indicate a mass loss.

### Setting Time

Setting times of AH Plus and EndoSequence were significantly reduced after heating (p<0.05). On the other hand, BioRoot-RCS and Bio-C sealers had no significant reduction (p>0.05). Regardless of the heating, BioRoot-RCS had the shortest setting time, significantly different from AH Plus and EndoSequence (p<0.05).

### Flowability

The AH Plus sealer became significantly less flowable after heating (p<0.05). Heating did not affect the flowability of the other tested sealers (p>0.05). Regardless of the heating, the BioRoot-RCS had the lowest flow among the tested sealers.

### Solubility

The solubility of Bio-C after drying was -5.52% for unheated samples and -5.22% for heated samples. The solubility was higher than the ISO6876/ANSI57 recommendations for both experimental conditions, with no statistically significant difference between them (p>0.05). However, when hydrated and heated, the solubility of Bio-C was significantly lower in comparison with the hydrated and unheated samples (p<0.05). The solubility of EndoSequence BC Sealer was significantly higher (p<0.05) for the heated and dehydrated samples after 30 days of storage (Table 1). The solubility of AH Plus and BioRoot-RCS was not affected by heating (p>0.05). AH Plus had the lowest solubility (p<0.05) in all conditions when compared with the other tested sealers.

### Dimensional change

The heat did not affect the dimensional change of the sealers (p>0.05).

### pH value

The pH of the heated sealers showed no statistically significant changes (p>0.05), except for AH Plus, in which the pH increased from 6.85 to 7.26 during the 24-h period (p<0.05). In both subgroups (heated and unheated), the calcium silicate sealers promoted significantly higher pH values (p<0.05) than the epoxy resin-based sealer.

### SEM/EDS analysis

The results of the SEM and EDS analysis may be seen in Figures 1-4. SEM analysis revealed that heating promoted significant changes on the sealers’ surface (Figures 1 ,2,3,4,to 4, A to C: unheated sealers; and E to G: heated sealers), especially for EndoSequence (Fig 2, E-G) and BioRoot-RCS (Fig 3, E-G). There was an increase in the deposition of crystals on the sealers’ surface after heating, which turned them more irregular. In higher magnification (×2000), SEM showed structures similar to “flower petals” in the EndoSequence surface (Figure 2G), and glomeruli-like structures in the BioRoot-RCS surface (Figure 3G). EDS analysis (Figures 1,2,3,4, D: unheated sealers; and H: heated sealers) showed peaks of zirconium, silicon, and calcium, and also demonstrated that heating led to higher peaks of calcium, especially for EndoSequence (Figure 2, H).

## Discussion

The results obtained in this study demonstrated that heating affected several properties of the tricalcium silicate sealers. Then, the initial null hypothesis was rejected. For all tests, the sealers were heated to 100ºC for 1 min and kept at 37 ºC to simulate the clinical scenario during warm vertical compaction ^3^. The sealers were placed in plaster molds that had been immersed in water for 24 hours to simulate the root canal system moisture. Because of their hydraulic nature, moisture is required for the hydration of tricalcium silicate sealers, especially for the premixed versions ^9^.

Results from the present study indicate that heat did not affect the setting time of the Bio-C sealer. The setting time of the unheated Bio-C sealer (140 min) differed from that reported in a previous study (220 min) ^8^, even with the use of a similar methodology. This discrepancy may have been attributed to the type II plaster used to make the molds, compared to the type IV plaster used in the previous study, with the former being more porous and able to store more moisture. The setting time of BioRoot-RCS was not influenced by the heat and was within the range reported previously (55 min) ^16^. For the EndoSequence BC sealer, Aksel and colleagues reported an increase in setting time when the sealer was heated to 200 ºC for 30 s ^17^. Conversely, another study reported a small reduction in setting time when the sealer was heated at 100ºC for 1 min ^18^; their result was analogous to that identified in the present work. It is speculated that the high temperature is responsible for this behavior. As water from the mixed sealer (powder-liquid version) or dentin (premixed versions) evaporates during heat application, there is less moisture available for the hydration of tricalcium silicate or dicalcium silicate particles within the hydraulic sealers ^5^. This may be the reason for the decrease in the initial setting time of the EndoSequence BC sealer ^5^.

The flowability of the three tricalcium silicate sealers was not affected by heat application. The heated Bio-C Sealer showed a lower flow than the EndoSequence and was similar to the BioRoot-RCS. However, BioRoot-RCS has a flow value that is lower than that recommended by ISO 6876 (a diameter not less than 17 mm) ^15^. This occurred probably due to dehydration and degradation of some components. It is important to emphasize that the manufacturer recommends the use of BioRoot-RCS with a single cone or cold lateral compaction technique. This result is similar to what was reported on BioRoot-RCS in another study ^2^. However, Yamauchi et al. (2021) ^19^ showed a significant reduction from 23.8 mm to 9.0 mm in flow for EndoSequence after heating to 100 °C for 1 min. It seemed to be a drastic reduction compared to a similar study conducted by Antunes et al. (2021) ^6^ which found 29.41 mm in flowability after heating in the same parameters. Another study conducted by Aksel et al. ^17^ verified that BioRoot was greatly affected by heat application (exposed to 200 °C for 10 or 30s), which resulted in a significant reduction in flowability and increased viscosity.

Root canal sealers should have less than 3% solubility ^14^ ^,^ ^15^. Desiccated samples were used to assess the influence of humidity on the solubility of the sealers. When examined wet, Bio-C Sealer was less soluble after heating than without heating. However, the solubility in the dehydrated samples was higher than what was recommended by the two standards. This finding collaborated with the results of previous studies ^8^ ^,^ ^20^. Bio-C Sealer showed greater biocompatibility and bioactivity than AH Plus sealer ^21^. The solubility values of BioRoot-RCS and EndoSequence BC sealers, even after heating, were within the recommended solubility for sealers. Heating did not influence the solubility of BioRoot-RCS solubility irrespective of whether the samples were weighed wet or dry. However, a recent study reported otherwise ^17^; BioRoot-RCS lost more weight when heated to 200 °C for 30 s. The authors attributed this to the higher water content of BioRoot-RCS at baseline, which is 3.5-4 times more than EndoSequence BC sealer.

Bio-C Sealer expanded more than the recommended value (0.1%) after heat application ^14^. BioRoot-RCS shrank slightly after 24 hours but expanded slighter at 30 days. EndoSequence BC Sealer shrank slightly after 24 h and at 30 days, corroborating with the results reported in previous studies ^8^ ^,^ ^20^.

The alkalinity of a sealer may increase the pH value of tissue fluids, which is low in the presence of localized inflammation ^11^. For this reason, pH is considered an important chemical property of a root canal sealer ^22^. Heating did not alter the alkalinity of all the tested sealers which corroborates with previous publications ^3^ ^,^ ^4^ ^,^ ^6^. The high pH values of the tricalcium silicate sealers are related to the formation of Portlandite (calcium hydroxide) as a hydration product, which results in calcium and hydroxyl ions released ^6^ ^,^ ^23^.

It was possible to observe by SEM that heating promoted an increase in the deposition of crystals on the surface of the sealers, which turned them more irregular. The formation of calcium hydroxide crystals similar to “flower petals” in EndoSequence and glomeruli in BioRoot-RCS was noted by us. For some crystals, these formations are strictly parallel to the surface and grow like “flower petals” around the center point, sometimes reaching a perfect hexagonal shape. This highly organized structure was less visible in the unheated samples. Similar characteristics were observed for BioRoot-RCS by Kharouf et al. (2020) ^24^, however, the sealer was not heated. This deposition was less intense and occurred in the form of small dispersed crystals on the surface of Bio-C, however, still present in a significant amount when the sealer was heated. The heating of the bioceramic sealers probably accelerated the growth of calcium silicate hydrate (C-S-H) promoting the occurrence of a greater number of hydration nuclei with the typical deposition of calcium hydroxide crystals ^25^. The characteristic hexagonal shape of the crystals, as well as their layered organization, allow them to be identified as calcium hydroxide ^25^. This was confirmed by the EDS analysis, showing the presence of a high peak of calcium (Figures 1,2,3).

EDS analysis has been broadly used to determine the chemical elements present on the surface of materials ^3^ ^,^ ^4^ ^,^ ^6^ ^,^ ^11^. In the current study, it was observed that the silicate-based sealers showed peaks for zirconium, referring to their radiopacifiers, as well as for silicon and calcium, constituents of the tricalcium silicate hydrate matrix. Peaks for carbon were also detected in AH Plus, which corresponds to the organic matrix of the polymers. In addition, the BioRoot-RCS recorded a chlorine peak from the calcium chloride, a setting accelerator present in the material's liquid, which has also been demonstrated in the study by Siboni et al. (2017) ^17^ and Antunes et al. (2021) ^6^. EDS was also performed after a heat process in the sealers. When variations in heat occur, it is possible to observe higher peaks for calcium in EndoSequence and BioRoot-RCS.

The present study has some drawbacks that should be discussed. As with other laboratory studies, our research has limitations inherent to its methodological design. The use of controlled methods and standardized samples benefits the comparation among the tested materials, but also constitutes a limitation of physical-chemical tests, as may not represent the true clinical behavior of the evaluated sealers. The use of other specifications, like the ASTM C266 standard, would be suitable for the setting time evaluation of the sealers ^6^ ^,^ ^22^. However, the ASTM C266 is recommended for hydraulic binders, as Portland cement, used in civil construction, and it demands specimens with greater dimensions ^26^. In the present study, we followed the ISO 6876:2012 standard, which is specific for root canal sealers ^15^. Root canal sealers that require moisture have already been included in the ISO 6876:2012 standard due to the limitation of former methods which were not adequate for this type of sealers ^26^. In this case, plaster molds are obtained ^27^ to provide the necessary moisture for the cement hydration process. A more recent study (6) proposed combining the ISO 6876 standard for sample preparation with a more rational amount of material, and the ASTM C266, for the implementation of the test, which could be more appropriate.

Bio-C Sealer is a new sealer available on the market. As with all new bioceramic materials, this sealer requires further researches. Promising results such as a shorter setting time, alkalinization ability, and adequate flow and radiopacity were reported in a recent study ^8^. The main concern regarding Bio-C Sealer is its solubility, as it is not in accordance with the ISO or ANSI/ADA recommendations, although this sealer showed low volumetric change ^8^. Our results corroborate these findings. Also, our study demonstrated the high solubility of Bio-C Sealer, especially for the dehydrated samples. However, heating did not affect its solubility rates.

Finally, according to a recent literature review ^28^, some properties of tricalcium silicate sealers are optimistic and are close to those of an ideal root canal sealer criterion (proper handling characteristics, antimicrobial activity, good sealing ability, radiopacity, biocompatibility, and adhesion) ^28^. Corroborating with our study, they have adequate setting time and dimensional stability ^28^. However, their high solubility remains a negative feature ^28^. On the other hand, from a biological point of view, greater solubility may be associated with higher ions calcium release, and consequently, greater bioactivity ^28^. Although the present study did not test bioactivity, this is an important property of tricalcium silicate sealers and is claimed to be their greatest benefit.

Within the limits of the present study, it may be concluded that heat application had different effects on the physicochemical properties of tricalcium silicate root canal sealers. Heat application was associated with the decrease in the EndoSequence BC sealer setting time. Also, heating promoted significant surface changes in all sealers and it was possible to observe higher peaks of calcium for EndoSequence and BioRoot-RCS. Although the results of this study should be interpreted cautiously, as the laboratory conditions do not fully reflect applicable clinical situations, the use of tricalcium silicate sealers clinically in warm obturation techniques should be done carefully.
